# Differential Diagnosis of Uterine Leiomyoma and Uterine Sarcoma Using Magnetic Resonance Images: A Literature Review

**DOI:** 10.3390/healthcare7040158

**Published:** 2019-12-05

**Authors:** Ayako Suzuki, Masato Aoki, Chiho Miyagawa, Kosuke Murakami, Hisamitsu Takaya, Yasushi Kotani, Hidekatsu Nakai, Noriomi Matsumura

**Affiliations:** Department of Obstetrics and Gynecology, Faculty of Medicine, Kindai University Osakasayama, Osaka 589-8511, Japan; aoki@med.kindai.ac.jp (M.A.); mc672013@med.kindai.ac.jp (C.M.); kmurakami@med.kindai.ac.jp (K.M.); htakaya@med.kindai.ac.jp (H.T.); y-kotani@med.kindai.ac.jp (Y.K.); nakai@med.kindai.ac.jp (H.N.); noriomi@med.kindai.ac.jp (N.M.)

**Keywords:** uterine leiomyoma, uterine sarcoma, differential diagnosis, magnetic resonance images (MRI)

## Abstract

MRI plays an essential role in patients before treatment for uterine mesenchymal malignancies. Although MRI includes methods such as diffusion-weighted imaging and dynamic contrast-enhanced MRI, the differentiation between uterine myoma and sarcoma always becomes problematic. The present paper discusses important findings to ensure that sarcomas are not overlooked in magnetic resonance (MR) images, and we describe the update in the differentiation between uterine leiomyoma and sarcoma with recent reports.

## 1. Introduction

Uterine sarcoma is a malignant tumor of the uterine mesenchymal tumor that occurs in 0.7% of every 100,000 women and accounts for 3–7% of all malignant uterine tumors [1]. Among all types of uterine sarcomas, leiomyosarcoma (LMS) is highly malignant with an adverse prognosis, and even in Stage I, its 5-year survival rate is approximately 50%, with this figure reported to decrease less than 20% from Stage II onward [2]. Diagnosis and treatment of uterine sarcoma involve major problems, and it is well-known that establishing a definitive preoperative diagnosis of uterine sarcoma is difficult.

One of the challenges in diagnosing uterine sarcoma is differentiating it from degenerated uterine leiomyoma. Although magnetic resonance imaging (MRI) is often used to establish the proper diagnosis, currently, definitive differentiation between uterine sarcoma and degenerated uterine leiomyoma remains difficult. However, it is possible to extract a tumor suspected to be a sarcoma. The present paper discusses important findings to ensure that sarcomas are not overlooked in magnetic resonance (MR) images. It should be noted that sarcomas include LMS, low-grade endometrial stromal sarcomas (LGESSs), and high-grade endometrial stromal sarcomas (HGESSs) and exclude carcinosarcomas.

## 2. MR Imaging Sequences Required for Evaluation

- T2-weighted images (T2WI) (sagittal, axial section)
- T1-weighted images (T1WI) (sagittal, axial section)
- Diffusion-weighted image (DWI) (sagittal or axial section) and apparent diffusion coefficient (ADC) map
- Optional: Gadolinium contrast-enhanced images including fat-suppression T1W1 and dynamic MRI

## 3. Procedures for the Interpretation of MR images

In differentiating between uterine myoma and uterine sarcoma, what is required in the radiological interpretation of MR images is not the definitive diagnosis of uterine sarcoma but the following dictum: a mass that may possibly be sarcoma should not be overlooked. Hence, an accurate evaluation as to whether the identified mass can be diagnosed as MR images of typical uterine leiomyoma is required.

- In the T2WI, ascertain the mass signals. Are there mass signals in intermediate to high-signal areas? Typical uterine myoma is presented as low signals [3].
- In the T2WI, examine the mass borders. If the borders are ill-defined, this suggests infiltrative growth into the periphery, and sarcoma is suspected [3].
- In the case where the mass presents high signals in T2WI, then ascertain the signals in the T1WI. Hemorrhagic necrosis within the mass—required for the diagnosis of sarcoma—is shown as a faint high-signal area [3]. However, LGESS is typically presented as low signals in T1WI [4].
- Ascertain the contrast effect of the mass. While sarcoma shows a strong contrast effect from an early stage, in some cases, the contrast effect is insufficient, with an area presumed to be necrosis [4].
- In the DWI, is there a diffusion anomaly in the mass (apparent diffusion coefficient (ADC) values are low)? ADC values are low in a sarcoma with high cell density. Nevertheless, with this finding alone, it is difficult to differentiate this from myoma [3,4].

## 4. Modality of Evaluation Images

When clinically examining a patient considered to have uterine myoma, consistent with the internal (pelvic) examination, the diagnosis should be established using ultrasound (US) examination. If characteristic US findings for uterine sarcoma are observed, differentiation with myoma is possible. However, insufficient effective US findings, including Doppler echo images, for diagnosing sarcoma, are observed [5]. Thus, MRI scanning is essential for patients suspected with sarcoma.

## 5. MR Image Findings

**Figure 1:**
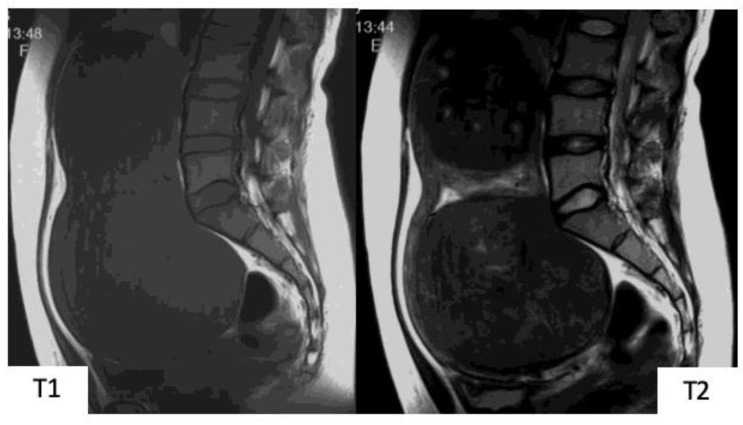
Typical images of uterine myoma in magnetic resonance images. In T1-weighted images, signals equivalent to or relatively lower than that of the normal myometrium can be observed. In T2-weighted images, signals that are clearly lower than those of the normal myometrium, with resection of almost all spherical masses with clearly defined borders, can be observed.

**Figure 2:**
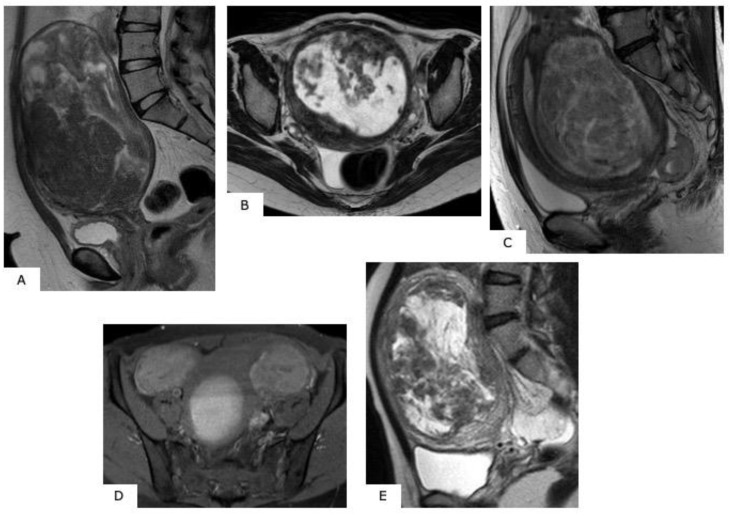
MR images of degenerated leiomyoma. These images always require a differential diagnosis from sarcoma. (**A**): hyaline degeneration (T2WI), (**B**): leiomyoma with cystic change (T2WI), (**C**): cellular leiomyoma (T2WI), (**D**): red degeneration (T1WI), (**E**): myxoid degeneration (T2WI).

An MRI scan is useful in differentiating between uterine myoma and sarcoma because a typical MR image for myoma exists. That is, uterine myoma shows a clear border, with a mostly spherical shape. In T1WI, there are more equivalent signals or relatively low signals compared to those for normal muscle layer, and in T2WI, mass is depicted in signals that are patently lower than those for normal muscle layer (Figure 1). Confirmation of an MR image enables the diagnosis of myoma. However, when the modification of degeneration is added to a myoma, a completely different image finding is presented than the above-described typical one (Figure 2). Subsequently, the differentiation of such a myoma from uterine sarcoma becomes problematic.

## 6. MR Images of Uterine Sarcoma

### 6.1. Characteristic MR Image Findings for Uterine Sarcoma

An understanding of MR image findings for sarcoma is necessary to differentiate uterine myoma from sarcoma. Here, findings suggestive of sarcoma are divided into the following two parts: LMS and endometrial stromal sarcoma (low-grade and high-grade).

### 6.2. Leiomyosarcoma

**Figure 3:**
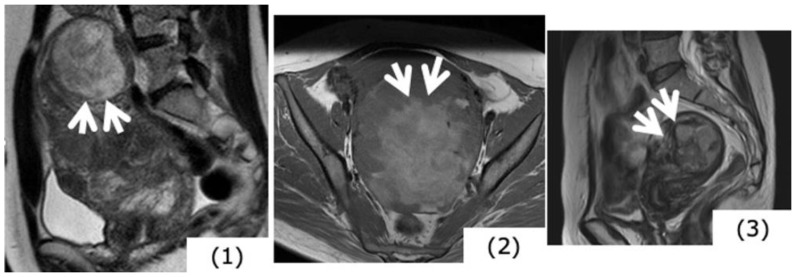
Magnetic resonance image findings considered to be characteristic of (specific for) uterine sarcoma. (**1**) High signals in T2-weighted images (T2WI) (case of uterine myoma): Mass in the fundus uteri has extremely high signal intensity (SI). (**2**) High signals in T1-weighted images (T1WI) (case of uterine sarcoma): There are mottled portions of high SI suggesting hemorrhage within the mass. (**3**) Ill-defined mass borders (case of uterine sarcoma, T2WI): Mass existing in the myometrium. High signals are presented in T2WI, and at the arrow portions, borders are ill-defined.

**Figure 4:**
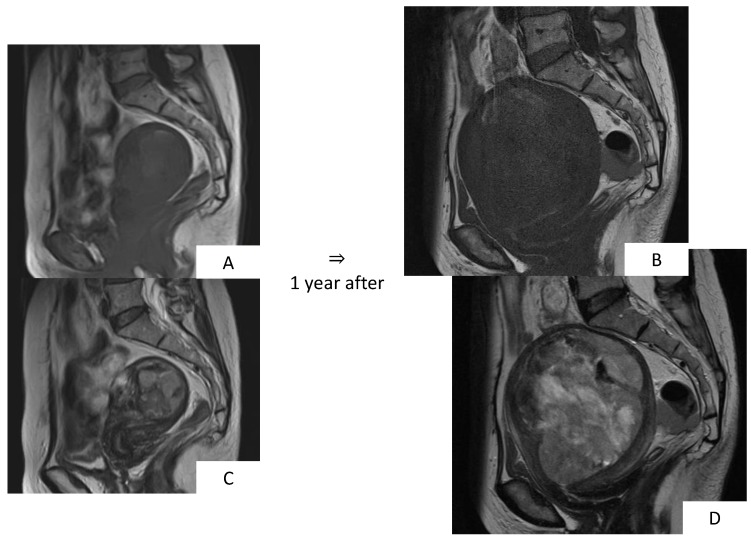
Magnetic resonance images of leiomyosarcoma. (**A**,**B**): T1-weighted sagittal sections, (**C**,**D**): T2-weighted sagittal sections. In the myometrium, in T2-weighted images, overall, there is high signal intensity with ill-defined borders; in T1-weighted images, there is mass accompanied by internal hemorrhage. Follow-up observation was selected for this case, with the diagnosis of uterine sarcoma 1 year thereafter.

**Figure 5:**
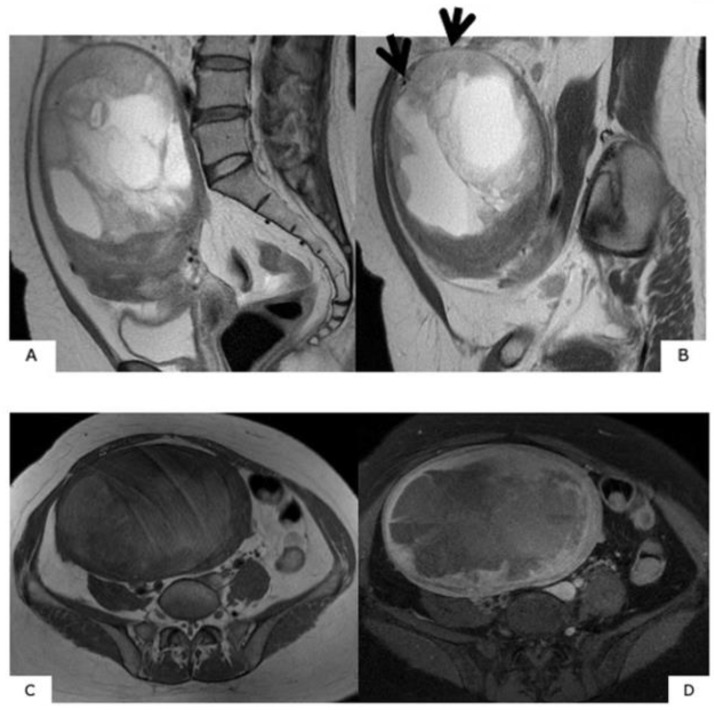
Magnetic resonance images of leiomyosarcoma (age, 49 years). (**A**,**B**): T2-weighted images sagittal sections, (**C**): T1-weighted axial section, (**D**): contrast T1 fat-suppression axial section. In the myometrium, heterogeneous high signals are presented with T2-weighted images, and with T1-weighted images, there is confirmation of mass showing faint high signals considered to be hemorrhage. In the contrast T1 fat-suppression image (**D**), a poorly contrasted area considered to be necrosis is found, while in (**B**), the fundus uteri side shows extremely thin myometrium, together with finding a portion considered to be extraserosal exposure, with ill-defined tumor borders (arrow).

LMS accounts for approximately one-third of all uterine sarcomas. The majority of LMS are observed in perimenopausal women aged 50–55 years, although 15% are noted in women aged less than 40 years. The following are well-known MRI findings for a suspected LMS: (1) high signals in T2WI and abnormal signals in DWI, (2) high signals in T1WI, and (3) ill-defined tumor mass borders (Figure 3) [3], with (1) showing high cell density in the mass and (2) suggesting hemorrhage within the mass and (3) an infiltrative growth of mass, a finding strongly suggestive of malignant tumor. In some cases, an image may show an exposure of the mass to extrauterine serosa, while in other cases, the mass has infiltrated to the surrounding normal myometrium and endometrium. This latter radiological interpretation should evoke special precaution. In contrast-enhanced images, a mass that shows an early period of heterogeneous, strong contrast effect is considered to be a tumor mass. However, the hemorrhagic necrosis portion of a mass—essential for the diagnosis of LMS—is considered to be an area where contrast effects are missing [4] (Figure 4 and Figure 5).

### 6.3. Endometrial Stromal Sarcoma (ESS)

**Figure 6:**
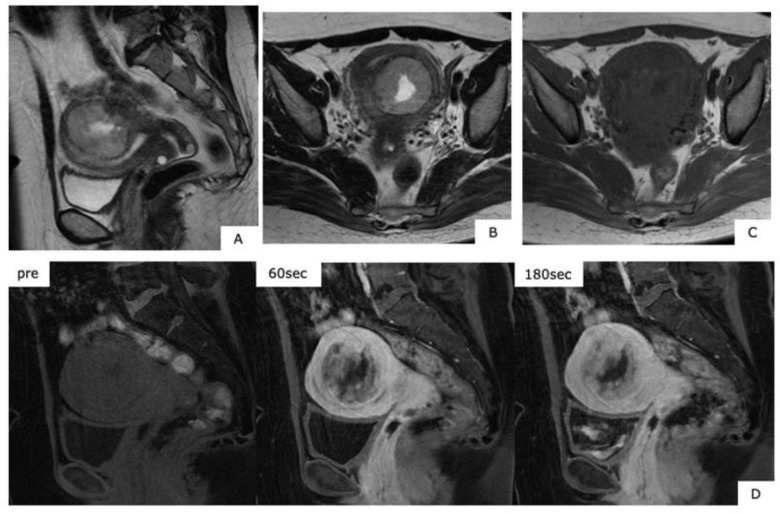
Magnetic resonance images of low-grade endometrial stromal sarcoma (age, 41 years). (**A**): T2-weighted sagittal section, (**B**): T1-weighted axial section, (**C**): T1-weighted axial section, (**D**): dynamic MRI contrast sagittal section. Within the uterine posterior wall myometrium, in T2-weighted images, there are mild high signals, with slow deep dyeing in the dynamic contrast-enhanced magnetic resonance imaging, with a finding of mass accompanied with internal necrosis. The T1-weighted images of C present faint high signals.

**Figure 7:**
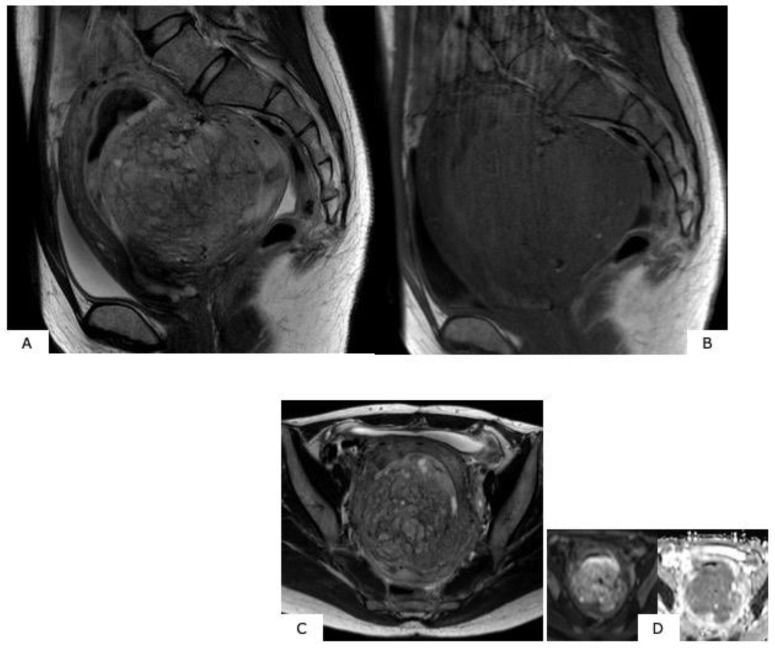
Magnetic resonance images of low-grade endometrial stromal sarcoma. (**A**): T2-weighted sagittal section, (**B**): T1-weighted sagittal section, (**C**): T2-weighted axial section, (**D**): diffusion-weighted image. Within the uterine posterior wall myometrium, in T2-weighted images, there are relatively high signals, and in T1-weighted images, a mass with ill-defined borders presenting low signals is observed. In (**A**) and (**C**), a low-signal band exists in the high-signal mass, presenting a “worm-like” finding that suggests that the low-grade endometrial stromal sarcoma is penetrating the normal myometrium while interposing itself intratumorally. Reduced diffusion is shown in (**D**).

ESS accounts for approximately 20% of uterine sarcomas and is classified as either low-grade (LGESS) or high-grade (HGESS) [6]. LGESS has a high incidence among premenopausal women aged 40–55 years and is characterized by its slow progress; most LGESS are diagnosed when restricted within the uterus (intrauterine) in Stages I and II [7,8]. In pathological images, in many cases, LGESS takes the form of a submucosal or intramural mass with ill-defined edges, which progresses such that it macroscopically appears that the myometrium is being retracted from the endometrium. In some cases, LGESS progresses in a worm-like manner intravascularly [7]. In MR images, the mass exists from the uterine cavity to the myometrium, with a typical presentation in T1WI as low signals and T2WI as heterogeneous high signals. In contrast-enhanced images, LGESS shows moderate and heterogeneous contrast effects. The well-known characteristic LGESS findings in MRI are the “worm-like” findings suggesting that the LGESS is penetrating the normal myometrium while interposing itself intratumorally and images that show worm-like interstitial and extrauterine extensions of multinodular mass [4] (Figure 6 and Figure 7). Diagnosis is simple when typical images are presented. However, in some cases, it is difficult to ascertain infiltrative growth, and due to the fact that LGESS characteristically occurs at younger ages, differentiating between a diagnosis of myoma and adenomyosis is frequently problematic.

The majority of HGESS occur in older women aged greater than 60 years [6,7]. Characteristic findings for HGESS are not reported in the literature. Many HGESS occupy the uterine cavity and grow, with a presentation of heterogeneous signals for both T1WI and T2WI. These are large masses that may develop into hemorrhagic necrosis and infiltration. In many cases, HGESS shows heterogeneous contrast effects, equivalent to or stronger compared to that of peripheral normal myometrium, considered a significant finding in differentiating HGESS from endometrial cancer [4,5,9].

## 7. Differentiating between Uterine Myoma and Sarcoma

There are various subtypes of uterine myoma, with degeneration also occurring. A variety of histopathological images serve as the basis for the variety in image findings, making differentiation from sarcoma difficult. For example, cellular leiomyoma presents high signals in T2WI due to its high cell density and shows reduced diffusion in DWI. Red degeneration accompanying pregnancy presents high signals in T1WI, and, in images, these are ascertained as tumor hemorrhage typical of sarcoma. Hence, there is an overlap in respective image findings for benign uterine myoma and malignant uterine sarcoma, indicating that there is a limitation in establishing an accurate diagnosis of sarcoma from image findings alone [3,4,5,9]. However, since findings suggestive of sarcoma are widely known, it is possible to “definitely ascertain masses that are potentially sarcomas” if they are not overlooked in radiological interpretations. Additionally, one should also consider careful assessment of patient information, including patient age, clinical symptoms, and examination findings, to establish an accurate diagnosis. Therefore, when relying on MRI scans, clinical data should also be communicated to radiologists. Prior communication of information is not only useful for radiological interpretations. Other responses are also then possible, including additional MR image sequencing, which aids in establishing the most accurate diagnosis.

## 8. Update on Differential Diagnosis of Uterine Myoma and Sarcoma

After the concerned parties were aware of the usefulness of MRI examination in diagnosing uterine myoma, several reports have been published that discuss “differential diagnosis of uterine myoma and sarcoma” using MR images, with consideration of progress and development for this examination in the future. Initially, several reports regarding the differentiation between uterine myoma and uterine sarcoma by combining a variety of clinical findings with findings of tumor configurations shown in MR images were observed. However, gradually, researchers became aware of the limitations of this approach alone [10,11,12,13]. Recently, there have been several reports of differentiation methods using DWI and ADC. In other words, these are methods that compare the respective biofunctions between uterine myoma and sarcoma.

In 2008, Tamai et al. reported that with DWI, uterine sarcoma and cellular leiomyoma presented high signals, while ordinary leiomyoma and degenerated leiomyoma were depicted in low signals. They further investigated the comparison of ADC values, reporting that this enabled the differentiation of LMS and degenerated leiomyoma. Tamai et al. also reported that there were cases of overlapping ADC values for the three items of ordinary leiomyoma, cellular leiomyoma, and LMS [14]. Subsequently, in 2009, Namimoto et al. combined tumor ADC values and the ratio of signal intensities in T2WI of the tumoral and normal myometrium, and they were able to reduce the overlap described above and reported moreover that this established the diagnosis of sarcoma with both for 100% sensitivity and specificity [15]. In 2013, Thomassin-Naggara et al. reported the diagnosis of sarcoma with 92.4% accuracy through a combination of T2WI signal intensity (SI), DWI with a b-value = 1000 s/mm^2^, ADC value (<1.23 × 10^−3^ mm^2^/s), and patient age [16]. Furthermore, in 2014, Sato et al. reported five cases of LMS with ADC values less than 1.1 × 10^−3^ mm^2^/s in 10 areas within the sarcoma. They then combined DWI SI with ADC value (<1.1 × 10^−3^ mm^2^/s) and differentiated myoma from LMS with high accuracy [17]. In 2015, Lin et al. found that while contrast-enhanced MRI resulted in a higher accuracy rate of diagnosis for sarcoma than DWI (*b*-value = 1000 s/mm^2^), the combination of DWI and ADC values (<1.08 × 10^−3^ mm^2^/s) established the diagnosis of sarcoma with accuracy rates equivalent to those of contrast-enhanced MRI. They reported that in patients with decreased renal function for whom contrast-enhanced MRI was difficult, discrimination with DWI was useful [18]. Recently, there have been several reports arguing the usefulness of DWI and ADC values [3,19,20,21,22]. However, in 2018, Kaganov performed a systematic review of past reports and found that a combination of T1WI and T2WI was effective, reporting that when there are high signals in T1 and high signals in T2, sarcoma can be diagnosed with 77.78% specificity. Kaganov found no significant association between ADC signals and sarcoma diagnosis [22]. Nagai et al. created the PREoperative sarcoma score (PRESS) using the four factors, such as age at operation, serum lactate dehydrogenase level, MRI findings (high signals in T1WI and/or heterogeneous signals in T2WI), and endometrial cytology findings and reported the usefulness of the PRESS. However, the point that neither DWI findings nor contrast-enhanced MRI findings are used should be noted in this analysis [23,24]. There is no doubt that signal intensities in T1 and T2WI, DWI, and contrast-enhanced MRI findings are each useful in the diagnosis of sarcoma. Nevertheless, as shown in Table 1, there is an overlap in MRI findings for myoma and sarcoma, which are not sarcoma-specific findings. Thus, currently, it is not the differentiation of sarcoma with high accuracy but surely “not to overlook even one single mass that may be a tumor” that is important.

## 9. Conclusions

We discuss the differential diagnosis of uterine myoma and sarcoma using MR images, including the most recent reports. While there are limitations regarding the differentiation between uterine myoma and sarcoma using MRI examination, MRI scans reliably extract cases for which the possibility of sarcoma cannot be excluded. According to the current data, it is essential to perform a detailed examination of MR image findings before surgery, and that mass that may possibly be sarcoma should be extracted, without any omissions.

## Figures and Tables

**Table 1:** Magnetic resonance image findings of ordinary leiomyoma, hypercellular leiomyoma, and leiomyosarcoma (modified from [3]).

	Ordinary Leiomyoma	Degenerated Leiomyoma	Cellular Leiomyoma	Leiomyosarcoma
Number	Multiple	Multiple	Multiple	Single
Well-Delinecated Margins	Yes	Yes	Yes	−
Endometrial Thinkening	No	–	−	Yes
Ascites	–	–	–	Yes
T2-Weighted Images	Hypointense	Hypo or Hyper Intense (Depending on the Type of Degeneration)	Intermediate Hypersignal	Hyperintense
T1-Weighted Images	Isosignal	Hypo or Hyper Intense (Depending on the Type of Degeneration)	Inosignal	Hypersignal
Diffusion-Weighted Images	Isosignal	Isosignal	Hypersignal	Hypersignal
ADC value <1.23 × 10^−3^ mm^2^/s	–	–	+	+
T1-Weighted Post-Gadolinium Chelate	Hypovascular heterogeneous	Hypovascular Heterogeneous	Homogeneous Progressive Filling-In	Early Heterogenous progressive fillng-in
